# Comparison of the plantar pressure distribution and mechanical alignment in patients with varus knee osteoarthritis following high tibial osteotomy

**DOI:** 10.1186/s12891-023-06603-7

**Published:** 2023-06-13

**Authors:** Ke Li, Feng-Long Sun, Heng-Bing Guo, Zhan-Jun Shi, Ran Yao, Hao Zhang

**Affiliations:** 1 Department of Orthopaedic Surgery, Nanfang Hospital, Southern Medical University, Guangzhou Road, Baiyun District, Guangzhou, Guangdong Province 510515 China; 2 Second Department of Orthopaedics, Capital Medical University affiliated Beijing Rehabilitation Hospital, Beijing, 100144 China

**Keywords:** High tibial osteotomy, Plantar pressure distribution, Lower limb alignment, Varus knee osteoarthritis

## Abstract

**Purpose:**

The changes in the lower limb alignment were vitally important after high tibial osteotomy (HTO). Therefore, the purpose of present study was to analyze the characteristics of plantar pressure distribution after HTO, and to investigate the effect of plantar pressure distribution on postoperative limb alignment.

**Methods:**

Between May 2020 and April 2021, varus knee patients undergoing HTO were evaluated in the present study. The peak pressure of plantar regions, medial-lateral pressure ratio (MLPR), foot progression angle (FTA), anteroposterior COP (AP-COP), lateral symmetry of COP (LS-COP), and the radiographic parameters were evaluated preoperatively and at the final follow-up. Compared among the slight valgus (SV), moderate valgus (MV) and large valgus (LV) groups at the final follow-up, the peak pressure of HM, HC and M5 regions, and the MLPR were compared; the Knee Injury and Osteoarthritis Outcome Score4 (KOOS4) including four subscales, and the American of orthopedic foot and ankle society (AOFAS) were evaluated.

**Results:**

The WBL%, HKA and TPI angle changed significantly after HTO (*P* < 0.001). The preoperative group exhibited a lower peak pressure in the HM region (*P* < 0.05) and higher peak pressure in the M5 region (*P* < 0.05); the pre- and postoperative groups exhibited a lower peak pressure in the HC region (*P* < 0.05); the rearfoot MLPR was significantly lower and LS-COP was significantly higher in the preoperative group (*P* = 0.017 in MLPR and 0.031 in LS-COP, respectively). Comparison among the SV, MV and LV groups, the SV group indicated a lower peak pressure in the HM region (*P* = 0.036), and a lower MLPR in the rearfoot (*P* = 0.033). The KOOS Sport/Re score in the MV and LV groups increased significantly compared with the SV group (*P* = 0.042).

**Conclusion:**

Plantar pressure distribution during the stance phase in patients with varus knee OA following HTO exhibited a more medialized rearfoot plantar pressure distribution pattern than that before surgery. Compared with the small valgus alignment, a moderate to large valgus alignment allows patients to walk with a more even medial and lateral plantar pressure distribution, which is more similar to healthy adults.

## Introduction

Medial knee osteoarthritis (OA) is especially common in the presence of varus alignment which altering loading and kinematics pattern in the knee joint [1]. The knee OA patients with varus deformity are predisposed to develop more severe OA if the abnormal alignment of lower limbs is not corrected [2]. High tibial osteotomy (HTO) is a well-accepted joint preserving surgery for patients with varus knee OA by substantially shifting dynamic knee joint loads laterally and restoring natural knee kinematics [3]. The typical aim of HTO is the reduction in magnitudes of the knee adduction moment (KAM), an indicator of knee load distribution associated with structural and functional deterioration of knee OA [4], and thus to improve the abnormal gait patterns and reduce loading in the affected side [5]. The changes in the gait and loading pattern can also exert an effect on the pressure distribution underfoot [6].

Plantar pressure analysis is beneficial for identifying the problem areas of the foot, and has been strongly influenced by the comprehensive understanding of the biomechanics underfoot, the focus of which has been on solving different concerns in regard to the association between abnormal plantar distribution and lower limbs posture [7]. In prior research, abnormally plantar pressure is prone to develop, or further exacerbate foot symptoms and plantar injuries [7, 8]. Further, a suitable amount of alignment correction in HTO could also elicit a rapid change in knee-ankle joint mechanics, which could exert a beneficial effect on the load of the ankle joint concerned [6, 9]. If the relationship between postoperative limb alignment and plantar pressure distribution is identified, assessment of the plantar pressure distribution may be of clinical significance in determining a more suitable postoperative alignment, thereby delaying or even avoiding the development of foot and ankle disorders. However, up to the present knowledge, there is no existing study in which the characteristics of plantar pressure distribution in varus knee patients following HTO are evaluated, and the relationship between plantar pressure distribution and postoperative limb alignment remains unknown.

The purpose of the present study was to analyze the characteristics of plantar pressure distribution during the stance phase in varus knee patients following HTO, and to investigate the effect of plantar pressure distribution on postoperative limb alignment. The present authors hypothesized that plantar pressure distribution in varus knee patients following HTO was distinct from that in healthy adults and preoperatively, and a more even plantar pressure distribution would be obtained in the medial-lateral compartment in the case of valgus correction with a moderate to large postoperative alignment.

## Methods

### Study protocol

In the prospective cohort study, HTO surgery was performed at our hospital by an experienced surgeon with over 20 years’ experience. The study protocol and consent forms were approved by the Ethics Committee of the hospital (2020bkkyLW005). All study participants read and signed a consent form approved by the Institutional Review Board prior to participation. The study included 71 patients (71 knees) with medial knee OA following HTO between May 2020 and April 2021. The participants were excluded as follows: (1) neuromuscular or spinal disorders affecting the lower limbs, such as cerebrovascular disease and lumbar spinal stenosis; (2) pre-existing ankle deformities such as pes planus, pes cavus, or ankle OA; and (3) patients who could not stand stably for over ten seconds during the stance phase because of pain or discomfort in the knee joint. The inclusion criteria for HTO surgery were as follows: (1) patients with medial unicompartmental OA; (2) medial proximal tibial angle < 85°, normal mechanical lateral distal femur angle (87° ± 3°), varus deformity ≥ 5°; (3) Kellgren-Lawrence (K-L) grade ≥ II [10]; and (4) body mass index (BMI) ≤ 35 kg/m^2^. The exclusion criteria for HTO surgery were: (1) previous history of knee or ankle surgery; (2) limited knee motion ≤ 100° or flexion deformity > 10°; (3) rheumatic arthritis or other inflammatory arthropathies; (4) symptomatic OA of the lateral compartment; or (5) ligamentous laxity around the knee and ankle. During the research, two patients were excluded due to pre-existingpes cavus, and three patients who could not complete the plantar pressure analysis were also excluded based on the verification of two experienced orthopedic specialists. A total of 66 patients (66 knees) who met the criteria for HTO were enrolled. Thirty-fourage-matched participants who had no previous knee pain and no known medical conditions that would affect gait were retrospectively enrolled in the control group, and were only used to clarify the changes in plantar pressure distribution in healthy adults.

In the subgroup analysis after surgery, patients were classified into three subgroups according to the postoperative weight-bearing line ratio (WBL%) [11]. The slight valgus group (SV group) consisted of 22 patients in which the WBL passed the tibial plateau in the 50–55% area, the moderate valgus group (MV group) consisted of 22 patients with the WBL passing through the 55–60% area, and the large valgus group (LV group) consisted of 22 patients in which the WBL was transferred to the 60–65% area.

### Surgical procedures and rehabilitation

Surgeons used an operative technique that was described in previous research [12]. At the start, knee arthroscopy was performed to debride the joint cavity and degenerated cartilage when necessary. Subsequently, two Kirschner wires were used to mark the osteotomy site from the distal third of the tibial tuberosity to the fibular head. The osteotomy procedure was performed using an oscillating saw and bone chisel and located 10 mm laterally from the hinge. According to the preoperative plan, the hinge was weakened by the Kirschner wire and opened to obtain valgus alignment. The osteotomy site in the medial tibia was fixed using a π plate and locking screws. Quadriceps femoris isometric contraction training was performed one day after surgery. A gradual increase in weight bearing was allowed within six weeks for progressive recovery. Full weight-bearing training was permitted after six weeks postoperatively.

### Plantar pressure analysis

#### Data collection

**Fig. 1 A:**
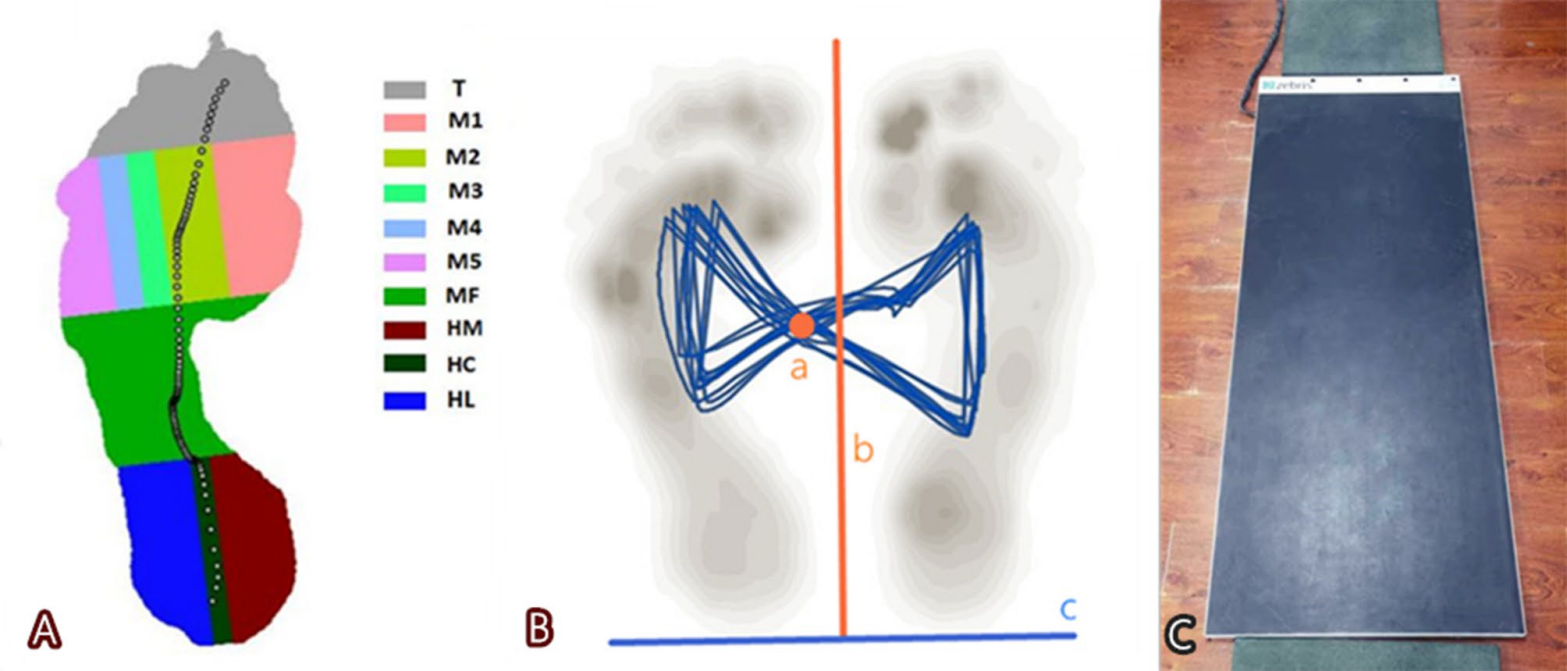
The peak pressure of the ten areas was shown according to plantar pressure analysis. Figure 1B, the anteroposterior COP (AP-COP) was the distance between the intersection point of butterfly plot (**a**) and the heel line (**c**); the lateral symmetry of COP (LS-COP, a positive value or negative representing transferring laterally or medially) was the distance between the point a and the centerline of graph (**b**). Figure 1 C, the pressure platform was shown to perform the plantar pressure analysis

Plantar pressure analysis was implemented preoperatively and at the final follow-up using the Zebris pressure platform (Zebris, Germany) at 100 Hz (Fig. 1C), which has been verified to have good reliability and repeatability [13]. The size of the force plate was 158 cm × 60.5 cm × 2.1 cm with a surface area resolution of 11,264 sensors on an area of 149 × 54.2 cm. Before data collection, the test system was calibrated according to each participant’s body weight. The force plate was located in the center of a carpet with the same external dimension serving as a 1.6 m-long platform, to record approximately three to five steps during the stance phase. For the participants, the peak pressure data was measured barefoot given their comfortable walking speed with a mean of 2.74 ± 0.46 km/hour.

For the patients with medial knee OA, the affected side was chosen; for the control group, the dominant foot was chosen [14]. The measurement procedure was repeated until three sets of valid data were acquired during the stance phase of gait.

#### Data processing

The Zebris software automatically divided the foot into the following 10 areas: medial heel (HM), central heel (HC), and lateral heel (HL), midfoot (MF), metatarsophalangeal joint 1(MTPJ 1), MTPJ2, MTPJ3, MTPJ4, MTPJ5, and toe (T) (Fig. 1A). In order to facilitate the overall comparison, the foot was classified into three parts given the anterior and posterior borders of the MF: forefoot (T and MTPJ1–MTPJ5), MF, and rearfoot (HM, HC, and HL). The foot midline (heel centroid to the forefoot between second and third metatarsal heads) divided the forefoot and rearfoot into two parts: the medial foot (M1, M2, and HM) and the lateral foot (M3–M5, and HL). The medial-lateral pressure ratio (MLPR) indicates the medial-lateral plantar pressure distribution in relation to the foot midline during the stance phase. The MLPR in the forefoot medial (M1, M2) / lateral (M3-5) and the rearfoot medial (HM) / lateral (HL) were recorded. The foot progression angle (FTA) was described as the angle between the foot long axis and the forward line, which could compensate for the increasing KAM of medial knee OA by toe-out gait. The Zebris software was equipped with the capability to calculate the center of pressure (CoP) parameters from the original data delivered by the platform, and the sampling duration for COP measurements was set to 60 s [15]. The anteroposterior COP (AP-COP) and the lateral symmetry of COP (LS-COP) were calculated according to the center coordinate of the COP trajectory [16] (Fig. 1B). AP-COP is the distance between the intersection point of butterfly plot (a) and the heel line (c); LS-COP (a positive value or negative representing lateral or medial transfer) is the distance between the intersection point of the butterfly plot (a) and the centerline of the graph (b). To assess the effect of postoperative limb alignment on plantar pressure distribution, the plantar regions showing the significant postoperative changes and MLPR were compared among the SV, MV and LV groups.

### Radiographic evaluation

**Fig. 2 A:**
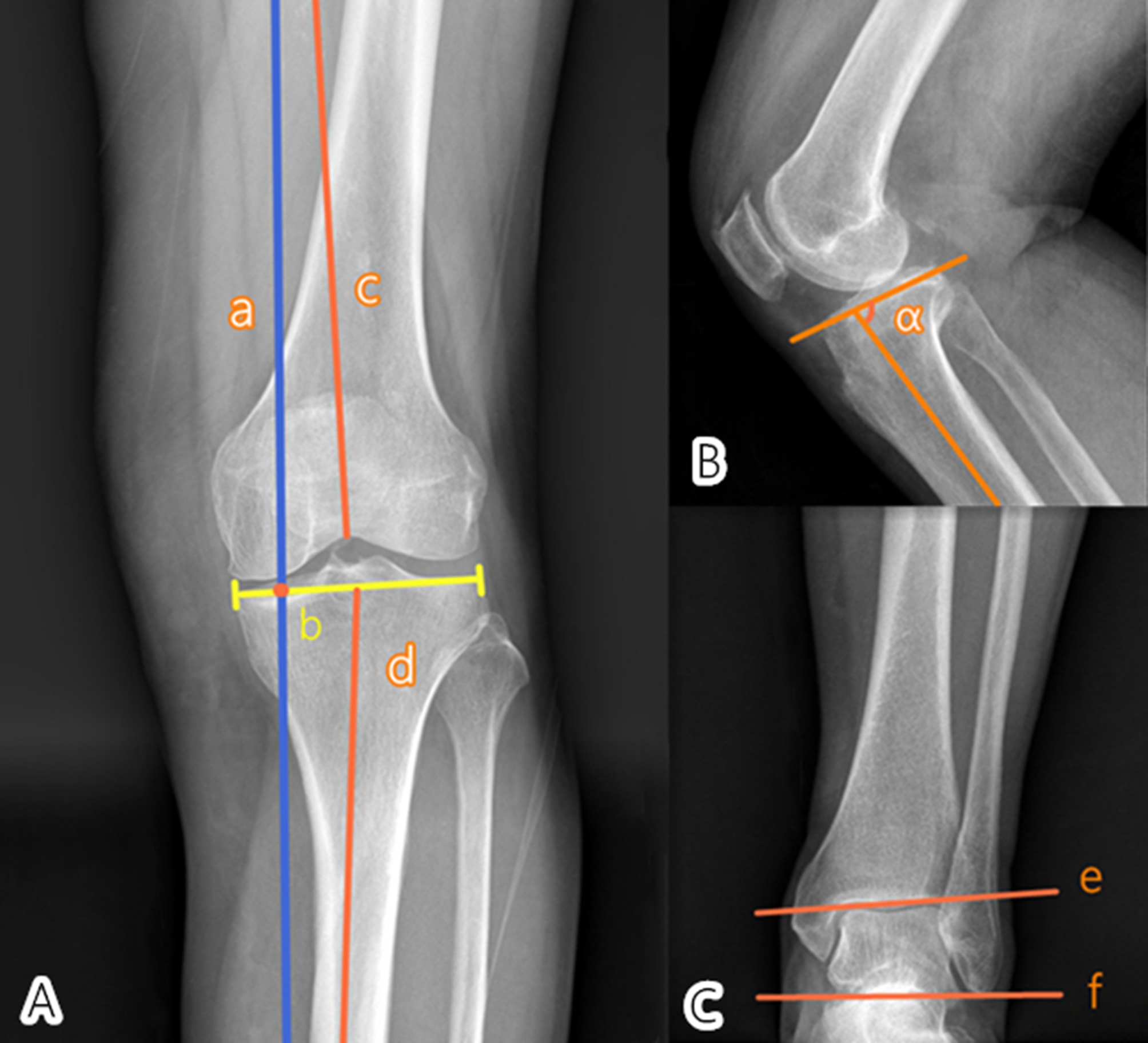
The weight-bearing line ratio (WBL%) was the proportion of the mechanical axis (**a**) passing through the tibial platform intersection point (**b**) and tibial platform width; The hip-knee-ankle (HKA) angle was the lateral angle between line C and D. Figure 2B, the posterior tibial slope (PTS) was the difference value between 90° and ∠α. Figure 2 C, the tibial plafond inclination (TPI) was the angle between the ground (**f**) and distal tibial plafond (**e**)

The radiographs were assessed on the basis of a picture archiving and communication system (PACS, PiView STAR, Seoul, Korea). Preoperative severity of knee OA was determined according to the K-L grade. Radiographic evaluations were performed using full-length anteroposterior weight-bearing radiograph of the lower limbs with the patella facing forward (Fig. 2). The WBL% (Fig. 2A) is the percentage of the mechanical alignment (a, the line from the femoral head center to the ankle center) passing through the tibial platform intersection point (b) and tibial platform width. The hip-knee-ankle (HKA, Fig. 2A) angle is the lateral angle between the center of the femoral head to the midpoint of the articular surface line in the distal femur (line c) and the midpoint of the tibial plateau to the midpoint of the ankle joint (line d) (a positive value or negative representing valgus or varus). The posterior tibial slope (PTS) in the lateral views of the knee joint (Fig. 2B) is the difference value between 90° and ∠α(the angle between the tibial plateau width and a line perpendicular to anatomical axis of tibia). The tibial plafond inclination (TPI, Fig. 2C) is the angle between the ground (f) and distal tibial plafond (e) (a positive value or negative representing lateral or medial inclination at the tibial plafond). All radiographic data were assessed preoperatively and at the final follow-up.

### Clinical evaluation

We used the Knee Injury and Osteoarthritis Outcome Score4 (KOOS4) and the American of orthopedic foot and ankle society (AOFAS) to evaluate the clinical outcomes of patients among the SV, MV, and LV groups. For the KOOS4, four subscales for pain, symptoms, sports/recreation (Sport/Rec), and quality of life (QOL) were calculated [17]. AOFAS scores were applied to assess the patient-reported foot and ankle outcomes [18]. Such measurements were obtained by two experienced investigators at the final follow-up.

### Statistical analysis

To evaluate the sample size, a preliminary study including 14 participants (HTO group, n = 7; control group, n = 7) was performed by means of G*power software (3.1.9.2) in an α-level of 0.05 and a power of 0.8. In the power analysis between HTO and control groups, 62 participants (31 patients per group) met the requisites, indicating a statistical significance. In the power analysis of the three subgroups after HTO, the sample size of 63 participants (21 patients per group) met the requisites. Therefore, we calculated the estimated sample size of 63 patients in the HTO group, and 31 patients in the control group.

Statistical analysis was performed using the SPSS 22.0 software (SPSS, Chicago, USA). All data were presented as mean ± SD. The independent-sample t-test, Chi-square test or Fisher’s exact test were used to assess the demographics data, while Paired t-test was used to compare the differences in pre- and postoperative radiographic measurements in the HTO patients. A repeated measures analysis-of-variance (ANOVA) with a post hoc analysis (Bonferroni’s test) was performed to compare the differences in the parameters of plantar pressure analysis and clinical results. A *P*-value < 0.05 indicated statistical significance. The measurement data was blinded for two measurements by two experienced independent observers with a time interval of at least two weeks. The intra- and inter-rater intraclass correlation coefficients (ICCs) were used for intra- and inter-observer reliability (ICCs > 0.75: good repeatability, ICCs < 0.40: poor repeatability).

## Results

The participant demographic data are shown in Table 1. No significant differences were observed in the demographic data between the HTO patients and the control group. The mean follow-up period after HTO was 18 months. The ICCs were > 0.90 (range 0.91–0.96) for all measured parameters indicating good agreement in terms of the reliability of measured measurements.

**Table 1:** Participant demographic data

Parameters	HTO patients	Control group	*P*-value
Number of patients	66	34	-
Age (years)	59.1 ± 8.6	57.8 ± 7.4	0.162
Male/Female	25/41	13/21	0.214
Weight (kg)	60.7 ± 10.5	64.3 ± 12.1	0.432
Body mass index (kg/m^2^)	23.5 ± 3.9	24.9 ± 4.3	0.373
Pre-op K-L grade 2/3/4	21/38/7	-	-
Follow-up period (months)	18.2 ± 6.8	-	-

Data are presented as the mean value ± standard deviation; HTO, high tibial osteotomy; K-L grade, Kellgren-Lawrence grade

The measured radiographic parameters are listed in Table 2. The WBL% translated significantly from 22.9%±10.8% preoperatively to 58.3%±11.6% postoperatively (*P* < 0.001). The HKA was corrected from − 6.9°±3.2° preoperatively to 2.8°±3.4° postoperatively (*P* < 0.001). The TPI angle changed significantly after HTO (6.7°±2.8° preoperatively, 0.9°±2.3° postoperatively, *P* < 0.001). However, no significant difference was found in the PTS after HTO.

**Table 2:** The radiographic outcomes in the knee and ankle joints before and after HTO

Parameters	Preoperative	Postoperative	*P*-value
WBL%	22.9 ± 10.8	58.3 ± 11.6	< 0.001*
HKA (°)	−6.9 ± 3.2	2.8 ± 3.4	< 0.001*
PTS (°)	9.1 ± 3.1	8.8 ± 2.9	0.278
TPI (°)	6.7 ± 2.8	0.9 ± 2.3	< 0.001*

Data are presented as the mean value ± standard deviation; HTO, high tibial osteotomy; WBL%, weight-bearing line ratio; HKA angle, hip-knee-ankle angle (a positive value or negative representing valgus or varus); PTS, posterior tibial slope; TPI, tibial plafond inclination; *P < 0.05

**Fig. 3:**
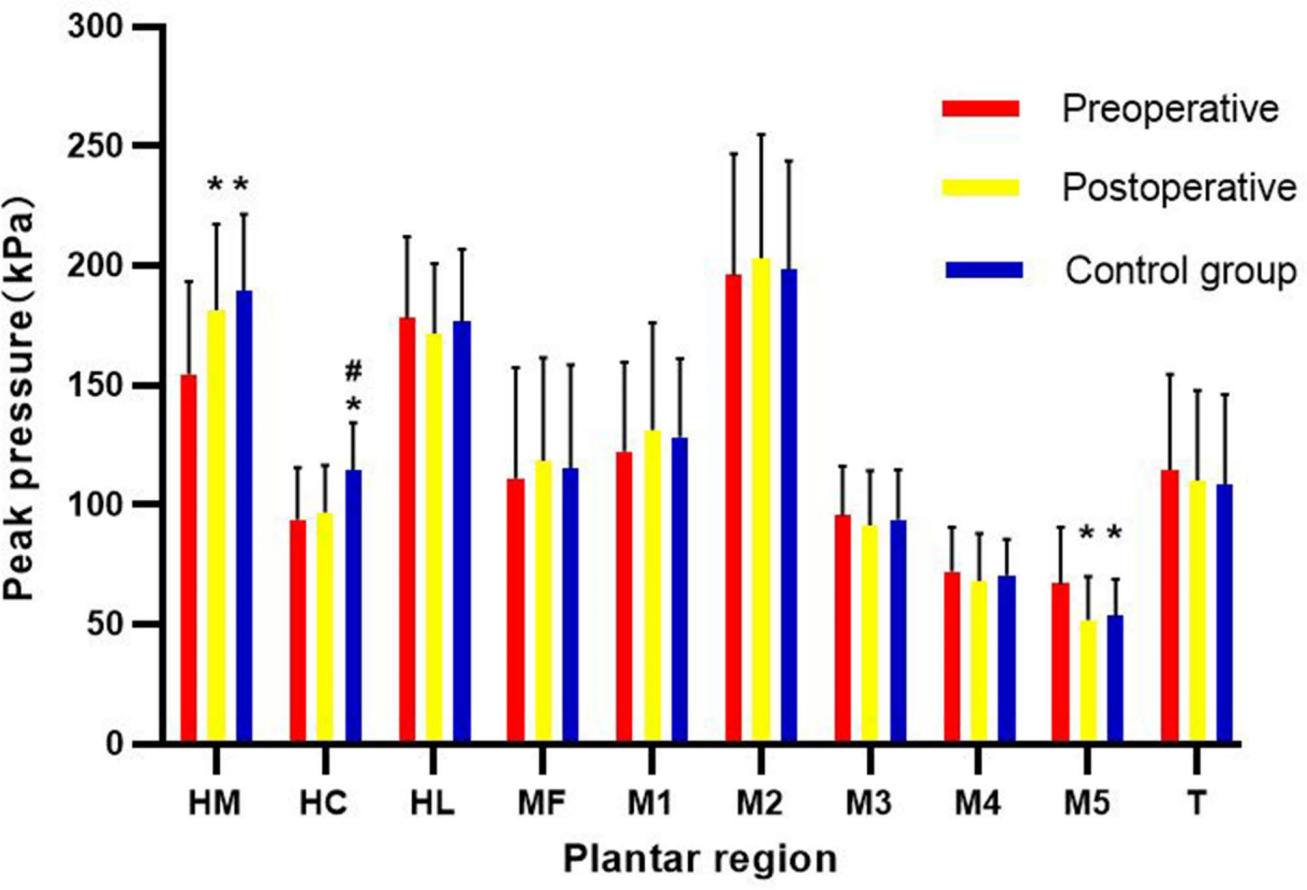
The peak pressure at each ten regions among the preoperative, postoperative and control groups. HM; medial heel; HC, central heel; HL, lateral heel; MF, midfoot; M, metatarsal; T, toe; *compared with the preoperative values, *P* < 0.05; ^#^compared with the postoperative group, *P* < 0.05

In comparing the peak pressure at each ten region among the preoperative, postoperative, and control groups (Fig. 3), the preoperative group exhibited a lower peak pressure in the HM region (*P* < 0.05) and higher peak pressure in the M5 region (*P* < 0.05), with no significant differences between the postoperative and control groups; the pre- and postoperative groups exhibited a lower peak pressure in the HC region (*P* < 0.05), with no significant differences between the pre- and postoperative groups; however, there were no significant differences in the rearfoot (HL region), MF, forefoot (T and MTPJ1–MTPJ5 regions) among the three groups.

In the assessment of MLRP, FTA, and COP among the three groups (Table 3), the rearfoot MLPR was significantly lower in the preoperative group than in the postoperative and control groups (0.83 ± 0.19, 1.03 ± 0.16 and 1.01 ± 0.21, *P* = 0.017), with no significant differences between the latter two groups; LS-COP was significantly higher in the preoperative group than the postoperative and control groups (6.3 ± 5.4 mm, -1.2 ± 6.8 mm and 0.2 ± 6.1 mm, *P* = 0.031), exhibiting no significant differences between the latter two groups; however, the forefoot MLPR, AP-COP and FTA showed no significant changes among the three groups.

**Table 3:** The outcomes in the MLPR, FTA, and COP among the preoperative, postoperative, and control groups

Parameters	Preoperative	Postoperative	Control	*P*-value
Forefoot MLPR	1.48 ± 0.19	1.58 ± 2.0	1.53 ± 0.15	0.146
Rearfoot MLPR	0.83 ± 0.19	1.03 ± 0.16*	1.01 ± 0.21*	0.017
FTA (°)	10.3 ± 4.1	9.2 ± 5.1	9.6 ± 4.7	0.461
AP-COP	137.8 ± 10.4	150.4 ± 11.7	146.5 ± 12.1	0.315
LS-COP	6.3 ± 5.4	-1.2 ± 6.8*	0.2 ± 6.1*	0.031

Data are presented as the mean value ± standard deviation; MLPR, medial-lateral pressure ratio; FTA, foot progression angle; AP-COP, anteroposterior center of pressure; LS-COP, the lateral symmetry of COP (a positive value or negative representing transferring laterally or medially); *compared with preoperative values, *P* < 0.05

In the comparison of the peak pressure of HM, HC, and M5 regions and the MLPR after HTO among the SV, MV, and LV groups (Table 4), the SV group exhibited a significantly lower peak pressure in the HM region than MV and LV groups (*P* = 0.036), but no significant difference in the HC and M5 regions; the MLPR in the rearfoot was also significantly lower in the SV group than that in the MV and LV groups (0.91 ± 0.17, 1.03 ± 0.19 and 1.06 ± 0.15, *P* = 0.033), with no significant differences between the latter groups; however, the MLPR in the forefoot showed no significant differences among the three groups.

**Table 4:** Comparison of plantar pressure distribution according to the different WBL% after HTO

Parameters	SV group	MV group	LV group	*P*-value
Number of patients	22	22	22	-
HM	167.4 ± 30.7	188.7 ± 27.6*	194.1 ± 28.2*	0.036
HC	94.3 ± 15.6	95.2 ± 18.9	93.7 ± 17.6	0.308
M5	54.8 ± 17.5	52.8 ± 19.2	47.6 ± 16.3	0.516
Forefoot MLPR	1.55 ± 0.16	1.57 ± 0.18	1.60 ± 0.13	0.179
Rearfoot MLPR	0.91 ± 0.17	1.03 ± 0.19*	1.06 ± 0.15*	0.033

Data are presented as the mean value ± standard deviation; WBL%, weight-bearing line ratio; HTO, high tibial osteotomy; SV, slight valgus; MV, moderate valgus; LV, large valgus; HM, medial heel; HC, central heel; M, metatarsal; MLPR, medial-lateral pressure ratio; *compared with preoperative values, *P* < 0.05

The results of the clinical examinations between the three groups are listed in Table 5. The KOOS Sport/Re score in the MV and LV groups increased significantly compared with the SV group (87.9 ± 17.5, 89.1 ± 13.9 and 77.2 ± 13.2, *P* = 0.042), and no differences were observed in the other KOOS subscores and AOFAS scores among the three groups.

**Table 5:** Comparison of the clinical results according to the different WBL% after HTO among the SV, MV, and LV groups

Parameters	SV group	MV group	LV group	*P*-value
Number of patients	22	22	22	-
KOOS4	87.4 ± 16.8	86.9 ± 11.6	88.1 ± 15.7	0.231
Pain	86.9 ± 15.2	90.4 ± 13.2	87.8 ± 11.6	0.198
Symptoms	86.3 ± 16.5	86.8 ± 12.7	89.1 ± 16.3	0.328
Sport/Re	77.2 ± 13.2	87.9 ± 17.5*	89.1 ± 13.9*	0.042
QoL	82.8 ± 12.4	84.7 ± 15.8	86.2 ± 13.4	0.395
AOFAS score	88.6 ± 10.9	91.1 ± 10.7	89.5 ± 9.3	0.423

Data are presented as the mean value ± standard deviation; HTO, high tibial osteotomy; WBL%, weight-bearing line ratio; SV, slight valgus; MV, moderate valgus; LV, large valgus; KOOS, Knee Injury and Osteoarthritis Outcome Score; Sport/Re, sports/recreation; QoL, quality of life; AOFAS, the American of orthopedic foot and ankle society; *compared with preoperative values, *P* < 0.05

## Discussion

In the present study, plantar pressure analysis was performed in varus knee patients undergoing HTO, and a more medialized plantar pressure distribution pattern than that before surgery was identified. In the moderate and large valgus groups, patients could walk with a more even medial and lateral rearfoot pressure distribution, being more similar to healthy adults. The results prove the original hypothesis. To the present knowledge, this is the first study that has demonstrated the characteristics of plantar pressure distribution, and evaluated the effect of lower limb realignment on plantar pressure distribution for varus knee patients undergoing HTO. The present findings contribute to a more comprehensive understanding of the postoperative characteristics of loading patterns in the plantar region, and provide clinically meaningful information for realigning the lower limbs following HTO.

In the medial knee OA, the mechanical axis of the lower limbs crosses the medial side of the knee joint, causing the change in the joint mechanical mechanics, and leading to an uneven distribution of the compressive load on the medial-lateral compartment of the knee [1, 19]. HTO is well-accepted joint preserving surgery aimed at realigning the mechanical alignment to the lateral compartment and redistributing the load on the knee joint [3]. However, the adjustment of lower limb alignment also has direct influences on the adjacent ankle joint, which has been demonstrated by a cadaver experiment conducted by Suero et al. [20] wherein various valgus realignments could significantly change the intraarticular ankle pressures and tibiotalar contact surface area. In the present study, the characteristics of plantar pressure distribution during the stance phase were evaluated, and valgus HTO was found to increase medial rearfoot pressure, reduce lateral forefoot pressure, and maintain midfoot pressures, while a lower peak pressure in central rearfoot was obtained compared with healthy adults. Given the redistribution of plantar pressure after HTO, such results indicate that varus knee patients following HTO exhibited a more medialized plantar pressure pattern than those before surgery, while the peak pressure in the plantar region was not still restored to a normal weight-bearing status. Such change in plantar pressure distribution can be further explained by the combined effect of the alignment correction in the knee-ankle joint. Firstly, during the entire stance phase, the patient suffered from deficient muscle strength in the lower limbs, which contributed to knee OA structural and symptom progression. After the valgus correction, the knee symptoms were alleviated with a notably decreased KAM [21]. As a result, the heel contacting the bearing surface accepted more body weight during the initial ground contact of the hind foot [20, 22]. Additionally, the adjacent ankle joint will also inevitably be affected to adapt to the changes of mechanical mechanics in the knee joint. Previous studies have found that after correcting the varus deformity of lower limbs, the alignment of the ankle joint and hind foot has obvious compensatory changes, being beneficial in reducing the incidence of ankle arthritis and relieving hind foot pain [2]. In the present results, the ankle joint improved from a lateral inclination preoperatively to a neutral inclination postoperatively. At the same time, a more medialized and even plantar pressure pattern was shown by correcting the mechanical alignment to a new balance point. Thus, there was a contribution to counteracting the compensatory valgus alignment of the rearfoot in patients with media knee OA [2, 23]. Such findings support those of previous studies indicating that the valgus-aligned HTO could significantly altered ankle pressure characteristics and improve the foot and ankle movement pattern in varus knee patients [22, 24].

The FPA modification in the toe-out gait may be a viable option for decreasing KAM and could be used as a treatment method for medial knee OA [25]. In the present results, although the valgus alignment could partially compensate the effect of toe-out gait on the KAM, the FTA scarcely changed after HTO, which could be attributed to several potential reasons. First, despite the large amount of the valgus correction, not all postoperative alignment may have achieved the large correction range of 60-65% in the tibial plateau width; second, FTA may have been influenced by other factors apart from the mechanical axis. For instance, knee flexion angle, lateral trunk tilt, muscle strength in lower limbs, and individual walking posture may also exert an influence on the FTA [26, 27]. In the analysis of the postural stability, the LS-COP was positioned significantly more centered for patients after HTO, and more similar to the healthy adults. The results indicate that patients undergoing HTO could maintain lateral balance more accurately during the stance phase than preoperatively. Such results may explain the superiority of mid-to long-term follow-up outcomes in patients following HTO [28, 29].

In the analysis of the effect in different lower limb alignments on plantar pressure distribution after HTO, only the rearfoot region exhibited a significant difference; however, no difference was found in the plantar pressure distribution of the forefoot among the three groups. Such results indicate that valgus lower limb alignment may exert an influence on the plantar pressure distribution of the rearfoot during the initial rearfoot ground contact in the gait cycle. Differing from the pressure distribution of the forefoot, which can be adjusted according to the load response when the pressure area is transferred from the rearfoot to the forefoot during heel off [30]. The most notable finding in the present study was that the patients with slight valgus alignment exhibited a more lateralized plantar pressure distribution pattern in the rearfoot during the stance phase than the patients with moderate and large valgus alignment who had a similar pressure distribution in the rearfoot region as in healthy adults. The present results indicate that moderate and large valgus correction could allow for a more even distribution of the medial-lateral plantar pressure during the stance phase. As reported in previous studies, an imbalance of plantar pressure is often related with knee-ankle pain during the stance phase and any region affected by pressure imbalance can often be injured [31]. As such, an appropriate increase in valgus correction is of clinical benefit in that the medial compartment of the knee joint is decompressed, while the plantar pressure is distributed more evenly, especially for varus knee patients with pre-existing foot symptoms. Surgeons performing HTO on patients with medial knee OA should also consider the effect of lower limb alignment on plantar pressure distribution in their clinical decision making.

In the present study, a more inferior KOOS Sport/Re subscale in patients with WBL50-55% was found, who were able to achieve a more lateralized plantar pressure distribution pattern, which did not correspond with the patients with WBL55-65% with a more even plantar pressure pattern. Such results are consistent with previous research [3]. A suitable valgus correction after HTO could achieve more normal knee kinematics; however, under-correction is associated with significantly worse functional recovery due to insufficient unloading in the medial knee joint. Additionally, no significant differences in the AOFAS score were found among the SV, MV and LV groups after HTO. Consequently, we could not confirm what degree of the plantar pressure distribution abnormalities might exert influence on the clinical results after HTO. The possible reasons for such results are the exclusion of patients with pre-existing foot symptoms or ankle deformities, and by excluding patients who were unable to complete the walking along the measuring platform to minimize the influence of clinical variables in the present study. Thus, in the future, further studies should be conducted to explore the characteristics of plantar pressure distribution in patients with pre-existing foot symptoms and how patients with pre-existing foot symptoms improve after HTO.

There were several limitations in the present study. First, the small number of enrolled patients and the wide variation of measured levels in plantar pressure between participants did not allow for a more in-depth prediction of the pressure threshold as an indicator for poor clinical outcome. Further studies need to be conducted with a larger sample size to clearly recognize a pressure threshold that would suggest that an individual is at risk of poor clinical results. Secondly, the change in distal tibial rotation after HTO has been widely reported [32], which might affect the ankle pressure characteristics and gait pattern. Further studies should be performed to assess the effect of distal tibial rotation after HTO on plantar pressure distribution. In addition, the follow-up period was relatively short, and further medium-long-term follow-up is essential to assess the change of plantar pressure distribution to draw a more comprehensive conclusion.

## Conclusion

Plantar pressure distribution during the stance phase in patients with varus knee osteoarthritis following HTO exhibited a more medialized rearfoot plantar pressure distribution pattern than that before surgery. Compared with the small valgus alignment, a moderate to large valgus alignment allows patients to walk with a more even medial and lateral plantar pressure distribution, which is more similar to healthy adults.

## Data Availability

The datasets generated and analyzed during the current study are available upon request from ZJS (orthopedicshi@126.com).

## Abbreviations

OA: Knee osteoarthritis
HTO: High tibial osteotomy
KAM: Knee adduction moment
K-L: Kellgren-Lawrence
BMI: Body mass index
WBL%: Weight-bearing line ratio
SV: Slight valgus
MV: Moderate valgus
LV: Large valgus
HM: Medial heel
HC: Central heel
HL: Lateral heel
MF: Midfoot
MTPJ: Metatarsophalangeal joint
T: Toe
MLPR: Medial-lateral pressure ratio
FTA: Foot progression angle
AP-COP: Anteroposterior center of pressure
LS-COP: Lateral symmetry of center of pressure
HKA angle: Hip-knee-ankle angle
PTS: Posterior tibial slope
TPI: Tibial plafond inclination
KOOS: Knee Injury and Osteoarthritis Outcome Score
AOFAS: American of orthopedic foot and ankle society
Sport/Rec: Sports/recreation
QOL: Quality of life
ANOVA: Repeated measures analysis-of-variance
ICCs: Intraclass correlation coefficients
